# Are current machine learning applications comparable to radiologist classification of degenerate and herniated discs and Modic change? A systematic review and meta-analysis

**DOI:** 10.1007/s00586-023-07718-0

**Published:** 2023-05-08

**Authors:** Roger Compte, Isabelle Granville Smith, Amanda Isaac, Nathan Danckert, Terence McSweeney, Panagiotis Liantis, Frances M. K. Williams

**Affiliations:** 1 Department of Twin Research, King’s College London, St Thomas’ Hospital Campus, 4th Floor South Wing, Block D, Westminster Bridge Road, London, SE1 7EH UK; 2 School of Biomedical Engineering and Imaging Sciences, King’s College London, London, UK; 3 Research Unit of Health Sciences and Technology, University of Oulu, Oulu, Finland; 4 Guy’s and St Thomas’ National Health Services Foundation Trust, London, UK

**Keywords:** Machine learning, MRI, LDD, Disc herniation, Modic change

## Abstract

**Introduction:**

Low back pain is the leading contributor to disability burden globally. It is commonly due to degeneration of the lumbar intervertebral discs (LDD). Magnetic resonance imaging (MRI) is the current best tool to visualize and diagnose LDD, but places high time demands on clinical radiologists. Automated reading of spine MRIs could improve speed, accuracy, reliability and cost effectiveness in radiology departments. The aim of this review and meta-analysis was to determine if current machine learning algorithms perform well identifying disc degeneration, herniation, bulge and Modic change compared to radiologists.

**Methods:**

A PRISMA systematic review protocol was developed and four electronic databases and reference lists were searched. Strict inclusion and exclusion criteria were defined. A PROBAST risk of bias and applicability analysis was performed.

**Results:**

1350 articles were extracted. Duplicates were removed and title and abstract searching identified original research articles that used machine learning (ML) algorithms to identify disc degeneration, herniation, bulge and Modic change from MRIs. 27 studies were included in the review; 25 and 14 studies were included multi-variate and bivariate meta-analysis, respectively. Studies used machine learning algorithms to assess LDD, disc herniation, bulge and Modic change. Models using deep learning, support vector machine, k-nearest neighbors, random forest and naïve Bayes algorithms were included. Meta-analyses found no differences in algorithm or classification performance. When algorithms were tested in replication or external validation studies, they did not perform as well as when assessed in developmental studies. Data augmentation improved algorithm performance when compared to models used with smaller datasets, there were no performance differences between augmented data and large datasets.

**Discussion:**

This review highlights several shortcomings of current approaches, including few validation attempts or use of large sample sizes. To the best of the authors' knowledge, this is the first systematic review to explore this topic. We suggest the utilization of deep learning coupled with semi- or unsupervised learning approaches. Use of all information contained in MRI data will improve accuracy. Clear and complete reporting of study design, statistics and results will improve the reliability and quality of published literature.

**Supplementary Information:**

The online version contains supplementary material available at 10.1007/s00586-023-07718-0.

## Introduction

Low back pain (LBP) is the leading contributor to disability burden globally and work disability [1]. LBP has a multifactorial aetiology, however lumbar disc degeneration (LDD) identified by magnetic resonance imaging (MRI) increases the risk of self-reported LBP up to 3.6-fold [2]. Although MRI should not be used in LBP diagnosis unless serious pathology is suspected [3], it is the current best available imaging tool to view soft tissue disorders, playing a role in LBP management and surgical treatment planning. MRI use throughout medicine is increasing, due to clinical benefits and improved patient safety. Demands on radiology departments have grown considerably with required radiological work hours outstripping those available [4]. MRIs take longer to read than other radiographic studies due to complexity and volume [4], with clinical spine MRIs, averaging 14–19 min to assess by an experienced radiologist [5]. The COVID-19 pandemic has exacerbated this pressure, hampering health care service provision including LBP diagnosis and treatment [6].

### Disc pathology identification and classification using MR imaging

**Fig. 1:**
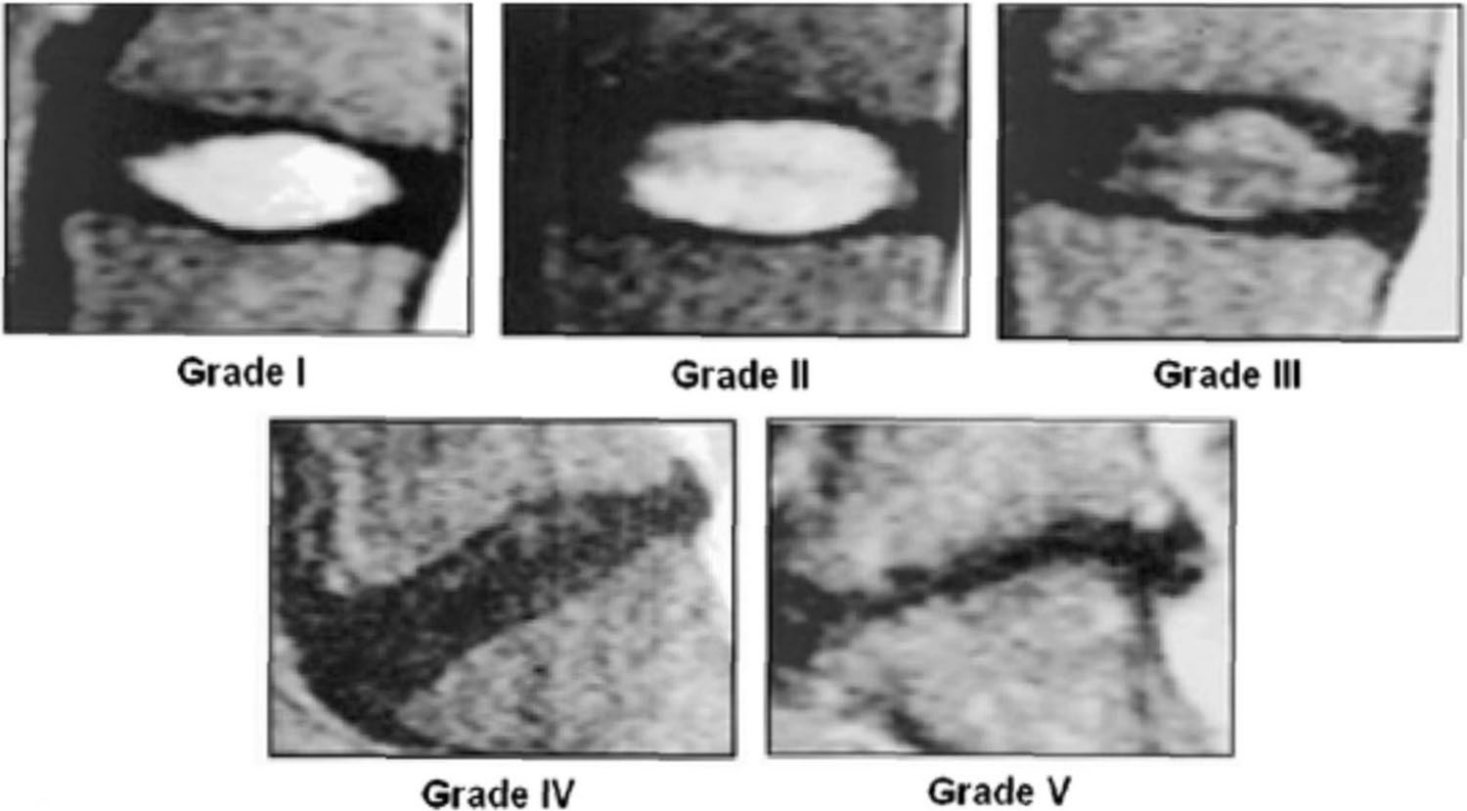
Pfirrmann grades on MRI. Image courtesy of Pfirrmann, C. W. A., Metzdorf, A., Zanetti, M., Hodler, J., & Boos, N. (2001). Magnetic resonance classification of lumbar intervertebral disc degeneration. Spine, 26(17), 1873–1878. 10.1097/00007632-200109010-00011

**Fig. 2:**
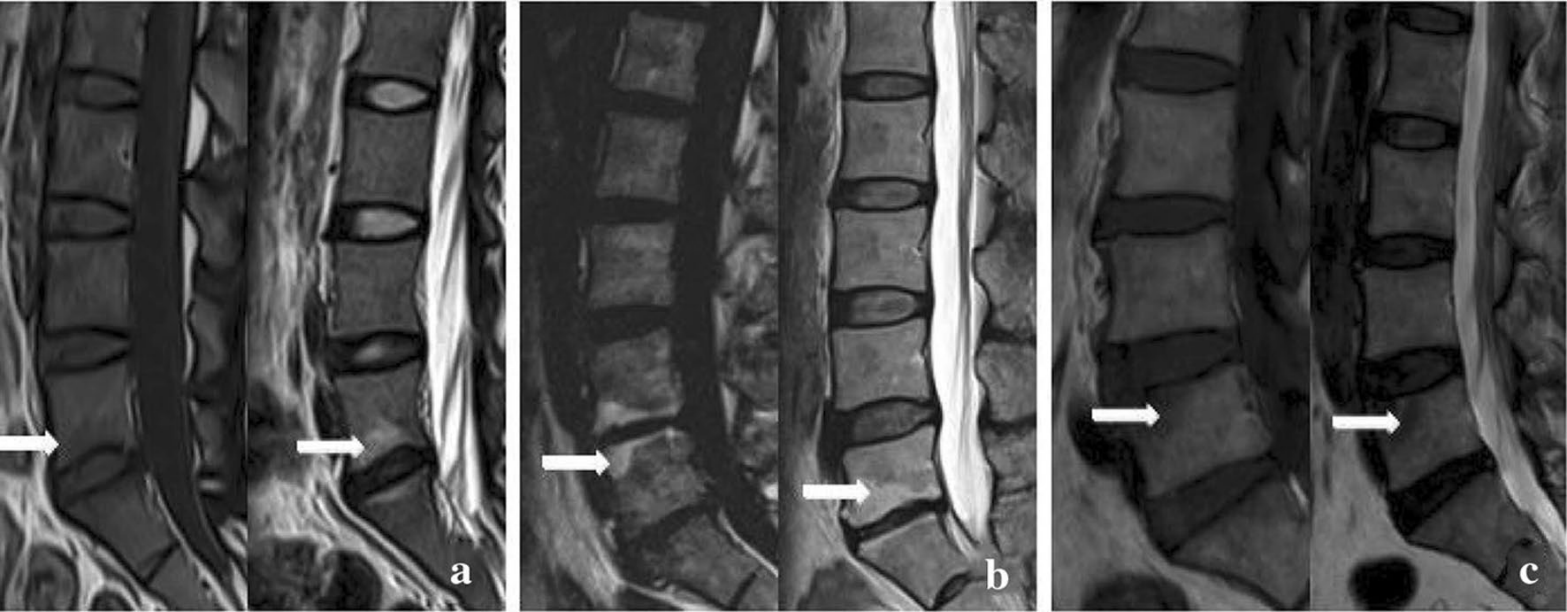
Modic change on MRI. Typical Modic changes. a Type I changes; b type II changes; c type III changes. Image courtesy of Chen Y, Bao J, Yan Q, Wu C, Yang H, Zou J. Distribution of Modic changes in patients with low back pain and its related factors. Eur J Med Res. 2019 Oct 9;24(1):34. 10.1186/s40001-019-0393-6. PMID: 31,597,571, PMCID: PMC6784341

Several grading systems are used to evaluate LDD [7–9]. It is commonly assessed with the 1–5 grade Pfirrmann scale [10]. Grade 1 healthy discs appear hyperintense, or bright on T2 weighted (T2W) MRI, due to their hydration. As discs dehydrate and degrade, image signal intensity is lost and at grade 5 badly degenerate discs appear black on T2W MRI, as depicted in Fig. 1 [11]. Shape changes are indicative: healthy discs are elliptical, whereas degenerated discs are flatter [12]. Modic change (MC) describes a bone marrow lesion in the vertebra adjacent to the bony endplate. MC type 1, associated with inflammation or increased water content in the endplate, is darker on T1 weighted (T1W) scans and brighter on T2W; types 2 and 3 show hyperintensity and hypointensity, respectively, on both T1W and T2W scans, as depicted in Fig. 2 [13–15]. Research to date has focused upon type 1, associated with advanced LDD, pain severity and worse prognosis [16]. The combination of LDD and endplate signal change is strongly associated with LBP [17, 18]. Capturing an accurate description of LDD on MRI presents several challenges. Distinguishing between the expected, age-related disc change in spine structures and abnormal or rapid degeneration that might lead to pain symptoms is difficult [19]. One study reported degenerate discs in 96% MRIs from 80 + year olds, who were not experiencing back pain [20]. There is high inter-rater variation between intervertebral disc pathology diagnoses and gradings [21–23]. The Pfirrmann scale only has moderate inter-rater agreement [24, 25] and can be difficult to use, with failures to distinguish early signs of degeneration [26]. The term disc bulge—whilst commonly used, lacks standardization and can be confusing, leading to poor communication between medical professionals [25].

### Machine learning

Machine learning (ML) developments offer a standardized approach and may detect patterns that have so far evaded human radiological enquiry. Further artificial intelligence (AI)-enabled solutions could improve the efficiency of reading scans [27] and accurately depict degeneration and other disc pathologies, thereby assisting clinicians and radiologists with correct diagnoses. Considering the shortfalls described above, simply to match human grading performance would not be optimal—ML may one day extend beyond current grading schemas. Several different ML approaches are currently used for automated reading of MRI scans [28] and inevitably their effectiveness needs to be evaluated. Deep learning (DL), algorithms use multiple layers of interconnected processing units able to extract and compile complex features from an input. Advantages to spine imaging such as standardized coding of defined phenotypes, improved accuracy of reading will improve research and patient care. Automated object recognition and classification for a broad range of spinal pathologies include successfully segmenting vertebral bodies on MRI [29–35]. Progress has been made in detection and classification of spinal deformity [36, 37] and high-performance ML models predict success or failure of spinal operations and postoperative complications [38, 39]. Early detection of pathological compositional rather than structural disc changes—has been demonstrated by comparing T1ro and T2 MRI relaxation times, important to note when there is a dearth of early process diagnostic tools [26]. Columbo software has recently been granted a world-first FDA clearance for their spine MRI-reading software [40]. While there are many encouraging reports of high accuracy models compared to human radiologists, there has been few replication studies which formally test algorithm validity. Along with replication trials, systematic, robust comparisons and evaluation of performance metrics that classify disc degeneration, herniation, budge or Modic change are needed. The aim of this review is to determine the ability of current ML technology for the classification of degenerate, herniated and bulged discs and Modic change. Successful ML models offer the exciting potential of real change for spine radiology, yet this promise is tempered by practical obstacles. Radiology departments will need to purchase special processing hardware (such as graphics processing units) to utilize algorithms yet may lack negotiating power to purchase at best price [41]. Rapidly improving technology presents the obvious threat of new ML assets being quickly out-of-date. Data and concept drifts need to be monitored as they can significantly undermine model performance in real-world settings [41, 42]. Models need to be not only accurate and reliable to be implemented clinically, but they need also regulatory approval. The beneficial, cost-effective implementation of ML technology in routine clinical practice goes well beyond the development and validation of software.

### What this review will add

AI-enabled applications are increasing in use throughout medicine. ML models reading MRI could save radiologists time and potentially surpass human diagnostic or prognostic accuracy. In clinical settings, an algorithm must be cost- and time-efficient, reproducible, offer standardized outcomes that are user-friendly and easy to integrate into approved picture archiving communication systems (PACS) software. ML is new in medicine and the contribution to spinal MRI reading depends upon how effectively and reliably detect it can classify and grade disc degenerative conditions including herniation, bulge and Modic change. The primary aims of this review and meta-analysis are to identify (1) if one model or software approach performs consistently well in identifying lumbar disc degeneration, herniation, bulge and Modic change, (2) if any MRI diagnostic tool is more amenable to ML and (3) document limitations of current ML applications.

## Methods

**Fig. 3:**
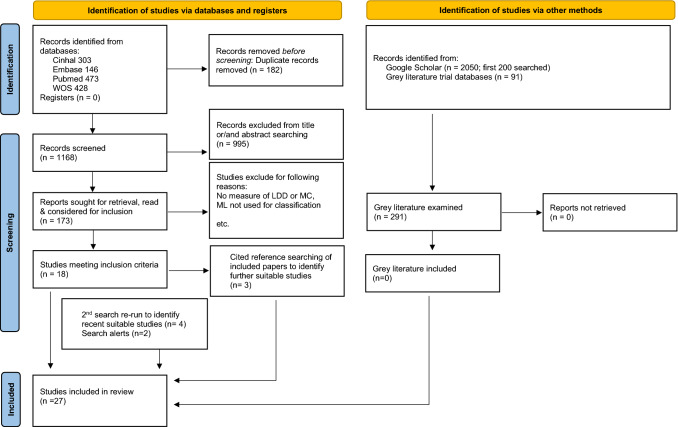
**Flow diagram based on the PRISMA statement**. PRISMA flow diagram: Are current machine learning applications comparable to radiologist classification of degenerate and herniated discs and Modic change?

A systematic review protocol was developed in accordance with PRISMA guidelines. The review was registered with PROSPERO (CRD42021261659) on 13th of July 2021 and is accessible (https://www.crd.york.ac.uk/prospero/). Four electronic databases were searched: Cihal, Embase, PubMed, Web of Science (including Medline) on 18th June 2021. Search terms, commands and outputs for databases are shown in Supplementary 1 (S1). Grey literature search details in S2. Details of article search results shown in PRISMA flow diagram (Fig. 3).

Studies were included if they met the following criteria: (1) original research articles (2) used ML algorithms to diagnose or measure LDD, herniation, bulge or Modic change and (3) used MRIs performed in adult humans. Abstracts, comparison trials or observational studies, detailing relevant findings or validation research for established algorithms were included. No date or language limits were set. Exclusion criteria specified case reports, editorials, letters, other meta-analyses or reviews. Only papers specifically using ML algorithms to identify or grade disc degeneration, herniation or bulge or MC were included. ML performance was to be compared to at least one human radiologist. Studies that focused on related conditions, for example spinal stenosis (only), were excluded. Studies using an imaging modality other than MRI were excluded. Algorithms that focused on image parameters necessary for but not sufficient to analyze LDD, for example segmentation (only) were excluded. This search was corroborated, and inclusion agreed by consensus of co-authors including a consultant radiologist (RC, IGS and AI). Eligible studies (*n* = 27) were identified and included in the review (Table 1). Study details such as prospective/retrospective design, algorithm development/validation and use of pre-processing or data augmentation were documented.

**Table 1:** Summary details of included studies

Author—date	Name of study	Disc pathology identified	# participants	# discs/ images	Design and algorithm/s used	MRI characteristics	Design/results comments
Athertya et al., 2019	Detection of Modic changes in MR images of the spine using local binary patterns	Development study assessing Modic change types 1 and 2 or ‘healthy’	100	500	Texture features extracted with local binary patterns. Compared classifiers kNN, NB, SVM & RT, with RF highest accuracy	T1W T2W sagittal plane 1.5 T	No pre-processing, so real-world validity, but aggressive data augmentation 10 MC1 10 cases trained as 160. MC increased frequency in older participants. MC type 1 more prevalent in females. Compared random train/test split with tenfold cross-validation. Contrast the use of data augmentation
Athertya et al., 2021	Classification of certain vertebral degenerations using MRI image features	Development study assessing Modic change severity (mild, moderate, severe) as well as chronicity (acute or chronic)	100	500	Texture analysis using the different local binary patterns. They compare the performance of random forest and SVM algorithms, reporting highest performance for SVM	T1W T2W STIRW sagittal plane 1.5 T	Same data as Athertya et al., 2019. Applied oversampling of cases (data augmentation) using synthetic data through SMOTE technique. They compare random train/test split with tenfold cross-validation. They also contrast the use of data augmentation
Beulah et al., 2018	Disc bulge diagnostic model in axial lumbar MR images using Intervertebral disc Descriptor (IdD)	Development study assessing disc bulge, disc herniation	93	675	Automatic axial segmentation followed by feature extraction and the linear combination of the different features. SVM was compared with kNN, decision tree and FFNN for binary disc bulge classification	T2W axial plane 1.5 T	Train/test split of the same number of cases and controls to avoid class bias. They assess disc bulge in a binary state. Posteriorly they extend the model to assess disc herniation
Beulah et al., 2021	Degenerative disc disease diagnosis from lumbar MR images using hybrid features	Development study assessing disc degeneration	93	558	Automatic sagittal segmentation followed by feature extraction of different texture features. SVM was compared with kNN, decision tree and FFNN for binary disc degeneration classification	T2W sagittal plane 1.5 T	Image pre-processing by filter application (e.g., maxima, thinning, opening). A hybrid model since training features include the signal intensity of MRI and invariant moments and Gabor texture features. tenfold cross-validation used in classification
Castro-Mateos et al., 2016	Intervertebral disc classification by its degree of degeneration from T2-weighted magnetic resonance images	Development study assessing Pfirrmann score	48	240	Semi-automatic segmentation followed by extraction of features to represent Pfirrmann definition (5 levels). NN classification performance compared (but not fully reported) with SVM and logistic regression	T2W sagittal plane 0.4 T	Novel extension of active contour models. Image pre-processing by pixel normalization and fuzzy C-means filtering. NN model selected includes swarm optimization. Images divided into test and training sets maintaining the proportion of different Pfirrmann levels in both sets
Ebrahimzadeh et al., 2018	Toward an automatic diagnosis system for lumbar disc herniation: the significance of local subset feature selection	Development study assessing disc herniation	30	210	Automatic segmentation, followed by feature extraction and feature selection. Classification of binary herniation performed with kNN, SVM and Deep learning	T2W used, sagittal plane and 1.5 T	Automatic segmentation based on spinal cord extraction previously to disc boundary definition. Features selected with feature decision tree. tenfold cross-validation used in classification
Gao et al., 2021	Automated grading of lumbar disc degeneration using a push–pull regularization network based on MRI	Development study assessing Pfirrmann score	500	2500	Automatic segmentation and classification of 5 grade Pfirrmann score using a CNN model	T2W sagittal plane 3.0 T	The CNN model consists of convolutional layers, pooling layers to extract image features and a fully-connected (FC) layers to perform classification. Push–pull regularization is used to enhance performance. Different CNN models were compared, Resnet-34 giving the best results
Ghosh et al., 2011	Composite features for automatic diagnosis of intervertebral disc herniation from lumbar MRI	Development study assessing disc herniation	35	175	Automatic segmentation followed by different feature extraction. Features are then combined, and different classifiers are used, including KNN, naive Bayes, and SVM	T2-SPIR sagittal plane 3.0 T	Automatic segmentation based on probabilistic models. Different features include raw, LBP (Local Binary Patterns), Gabor, GLCM (gray-level co-occurrence matrix), intensity and shape features. Dimensionality feature reduction using PCA and Linear Discriminant Analysis (LDA) and combinations were compared. tenfold cross-validation
Gong et al., 2021	Axial-SpineGAN: Simultaneous segmentation and diagnosis of multiple spinal structures on axial magnetic resonance imaging images	Development study assessing disc degeneration	62	169	Automatic axial segmentation of 4 spinal structures followed by a combination of CNN and other NN modules to perform feature extraction and disc degeneration (normal and abnormal) classification	T2W sagittal plane 1.5 T	Model includes a generator consisting of CNN and FC modules to combine spatially overlapping tissue information and extract features. Discriminator enhances generator performance. Diagnostic of output from different tissues. fivefold cross-validation
Grob et al., 2022	External validation of the deep learning 'SpineNet' for grading radiological features of degeneration on MRIs of the lumbar spine	External validation study assessing Pfirrmann score	882	4410	External validation consisting of patients with degenerative spinal disorders from 2 previous trials	T2W sagittal plane T—NR	Ground truth generated by a single radiologist. Inter-rater agreement between SpineNet and the radiologist demonstrated with weighted Kappa, CAA, Spearmans’ rank and Lin’s concordance correlation coefficients, and precision (positive predictive value), sensitivity (recall) and specificity
Han et al., 2018	Spine-GAN: Semantic segmentation of multiple spinal structures	Development study assessing disc degeneration	253	1818	Automatic segmentation, combination and binary classification of 3 spinal tissues. DL modules included CNN, atrous CNN and long short-term memory module (LSTM). Discriminator similar to Gong et al., 2021	T1W T2W sagittal plane 1.5 T	LSTM module integrates information on the different tissues and reduces overfitting. Discriminator helps increase segmentation and classification performance. fivefold cross-validation. Data collected from different centres, 2 radiologists established ground truth
Hashia et al., 2020	Texture features'-based classification of MR images of normal and herniated intervertebral discs	Development study assessing disc herniation	99	NR	Manual segmentation followed by different texture features extraction. Classification of binary herniation was performed with kNN, SVM and Back Propagation Neural Network (BPNN) on each set of features and performance was compared	T2W sagittal plane T—NR	Texture features set comprise features extracted by GLRLM, GLCM and GLDM texture analysis techniques, respectively. Data was split into train, validation and test sets for performance evaluation
He et al., 2017	Automated grading of lumbar disc degeneration via supervised distance metric learning	Development study assessing disc degeneration	93	465	HOG texture features were extracted from disc images. Features were used to classify discs as normal or slightly, marked or severe low disc degeneration by distance metric learning	T2W sagittal plane 1.5 T	Distance metric learning minimizes the distance between two distributions, in this case, the distribution of features and the distribution of levels on the grading. Feature distance was defined according to Mahalanobis distance. fivefold cross-validation
Jamaludin et al., 2016	Automatic Modic changes classification in spinal MRI	Development study assessing Modic Changes	444	4656 (endplates)	Automatic vertebra segmentation and alignment between T1W and T2W followed by histogram feature extraction. Features used to classify Modic change (no MC, type 1, 2 and 3) using SVM	T1W T2W sagittal plane T—NR	Features from T1W and T2W were joined by spatially-binned joint histogram of intensities, SJT. Data augmentation (43 transformations) was applied to 5 samples and performances of all transformations were mean. fivefold cross-validation
Jamaludin et al., 2017	SpineNet: Automated classification and evidence visualization in spinal MRIs	Development study assessing Pfirrmann score	2009	12,018	Automatic vertebra and disc segmentation followed by disc volume (3D) extraction. Pixels in the disc volume are used for classification of different spine conditions and scores (including 5-point Pfirrmann) in a CNN model	T2W sagittal plane T—NR	CNN model based on a modified version of VGG-M architecture. Deeper CNN architectures were tried but no differences were observed. Comparison of using 2D and 3D images shows in general better performance in 3D. Disc level adjustments were additionally implemented, and no improvement was observed. Different data augmentation strategies are used in the training step
Koh et al., 2012	Disc herniation diagnosis in MRI using a CAD framework and a two-level classifier	Development study assessing disc herniation	70	350	Manual segmentation and labelling followed by image subdivision and feature extraction. 4 different algorithms, including NN, k-means, SVM and a least mean square (linear) classifier, are combined in an ensemble classifier to diagnose herniation (binary)	T1W T2W sagittal plane 3 T	Image noise reduction applied to images. Feature vectors include the pairwise ratio of pixels corresponding to the spinal cord, vertebra and disc in the image subdivision. The ensemble classifier consists of a weighted agreement within the 4 different algorithms. Ground truth extracted from medical reports. Leave-out cross-validation used
Lehnen et al., 2021	Detection of degenerative changes on MR images of the lumbar spine with a convolutional neural network: A feasibility study	External validation study assessing disc bulge, disc herniation	146	888	External validation of Columbo software. Columbo is based on CNN architecture, and it can assess different spinal structures and classify different pathologies	T2W axial sagittal planes 1.5 T 3.0 T	Ground truth generated by a single expert reader. Subjects were patients with back pain. Images tested include 1.5 T or 3.0 T MRI. Comparison between software and reader was done by McNemar test and sensitivity, specificity, positive predictive value (PPV), negative predictive value (NPV), and accuracy
Lewandrowski et al., 2020	Feasibility of deep learning algorithms for reporting in routine spine magnetic resonance imaging	Development study assessing disc bulge, disc herniation	3560	17,800	Automatic axial and sagittal segmentation with 3D reconstruction. Classification using 4 class grading that includes bulge and herniation was accomplished using CNN	T1W T2W sagittal axial transverse planes T—NR	Ground truth from semi-supervised ML from radiologist reports trained using 5000 manually translated reports. Axial and sagittal MRI intersections were stacked and used as input for the CNN model. A final decision tree transforms the output of the classification into written reports. They used a random train/test split
McSweeney et al., 2022	External validation of Spine Net a deep learning model for automated grading of lumbar disc degeneration using the North Finland Birth Cohort	External validation study assessing Pfirrmann score, Modic change	684	3420	SpineNet external validation. Validation in a 4-point modified Pfirrmann grade and binary MC	T2W sagittal plane 1.5 T	Participants from a population birth cohort. 3 expert readers for Pfirrmann and 2 for MC scans. A subset of LBP subjects to match the training profile was separately evaluated. Lin's CC, Cohen's K, MCC, sensitivity, specificity and accuracy were reported
Niemeyer et al., 2021	A deep learning model for the accurate and reliable classification of disc degeneration based on MRI data	Development study assessing Pfirrmann score	1599	7948	Manual disc segmentation and labelling. Disc images were used to train CNN-based model to predict Pfirrmann score (5-point and extended 13-point score)	T2W sagittal plane 1.5 T 3.0 T	CNN used was modification of VGG-16 architecture. Ground truth from single radiologist. Extended fractional Pfirrmann score to account for between-grade presentations. Comparison between the classical and extended version reported. Data augmentation (fourfold) by rotation the training set. tenfold cross-validation
Nikravan et al., 2016	Toward a computer-aided diagnosis system for lumbar disc herniation disease based on MR images analysis	Development study assessing disc herniation	30	210	Automatic segmentation and disc labelling followed by intensity and shape feature extraction. SVM and neural networks were used for a binary herniation classification	T2W sagittal plane 1.5 T	Segmentation is based on Otsu thresholding and extraction of the spinal cord, followed by disc alienation and boundary definition. Random train/test split used
Oktay et al., 2014	Computer-aided diagnosis of degenerative intervertebral disc diseases from lumbar MR images	Development study assessing disc degeneration	102	612	Automatic segmentation of disc followed by different feature extraction. SVM classifier is used to classify binary disc degeneration	T1W T2W axial sagittal planes 1.5 T	Segmentation of discs were performed using active appearance models (AAM). Features include intensity, texture, whole shape, and context features. Each feature is tested by itself and in combination. Using all features gave best performance. fivefold cross-validation used
Pan et al., 2021	Automatically diagnosing disk bulge and disk herniation with lumbar magnetic resonance images by using deep convolutional neural networks	Method and development study assessing disc bulge	500	3555	Automatic axial and sagittal segmentation and next disc classification was performed using CNN modules. Classification levels comprised normal, bulge or herniated disc	T2W axial plane 3.0 T	3 CNN models were used to locate vertebral bodies, define intervertebral discs and classify the images. Classification used ResNet-101. Classification performance was assessed level-wise. They performed fourfold cross-validation
Su et al., 2022	Automatic grading of disc herniation, central canal stenosis and Nerve root compression in lumbar MRI diagnosis	Development study assessing disc herniation	1015	15,254	No segmentation used. Feature extraction and the following 4-point herniation classification was performed using deep learning architectures. 2 additional pathologies were also assessed. CNN ResNet-50 was used for feature extraction and FC layers for classification	T2W used, axial plane and 3.0 T	Ground truth by 2 readers with a 3rd one to solve disagreement. Participants were patients with back pain. Data augmentation by random rotation and cropping training data. Random training, validation and test splits on training data
		External validation study assessing disc herniation	100	1273	External validation of the model using patients from another hospital	T2W axial plane 3.0 T	
Sundarsingh et al., 2020	Diagnosis of disc bulge and disc desiccation in lumbar MRI using concatenated shape and texture features with random forest classifier	Development study assessing disc bulge	63	378	Automatic segmentation followed by different feature extraction and final disc bulge classification using random forest	T2W sagittal plane 1.5 T	Classification classes include normal, bulge and desiccated discs. Shape features (HOG) and texture features (LS-RBR) are combined and/or compared with combination giving a better performance. Random train and test split were used
Tsai et al., 2021	Lumbar disc herniation automatic detection in MRI based on deep learning	Development study assessing disc herniation	168	714	Automatic segmentation and classification of 4-grade bulge and herniation grading on normalized MRI. CNN architecture used after normalization	T2W sagittal plane 1.5 T	CNN is based on YOLOv3 (DarkNet-50) model. Grading includes bulge, protrusion, extrusion and sequestration extracted from clinical reports. They use different amounts of data augmentation to assess the level of over and underfitting and compare their performances. They use a random train/validation/test split. Performance measures used were not following standards
Zheng et al., 2022	Deep learning-based high-accuracy quantitation for lumbar intervertebral disc degeneration from MRI	Development study assessing Pfirrmann score	1051	5255	Focused on automatic segmentation with no classification but quantitative measurements of signal intensity and shape features. Features then regressed against different LDD scores and demographics	T2W sagittal plane 1.5 T	Segmentation based on CNN (BianqueNet – ResNet101). From disc segment, the signal-intensity difference (ΔSI), average disc height (DH), disc-height index (DHI), and disc height-to-diameter ratio (HDR) were calculated. These measurements were then correlated to Pfirrmann scores and additionally with different age ranges, sex and the disc level. No actual classification was performed with AI

Articles were tabled according to the PROBAST tool [43] to summarize the design of each study, assess risk of bias and determine the applicability of included models (S10). Studies that did not use standard disease definitions or did not report standard participant details or recruitment numbers or those failing to report variance statistics were ranked either *unclear* or *high* risk of bias.

### Meta-analysis

Studies were grouped according to classification measures. Groups included Pfirrmann and MC grades and binary or numerical LDD, herniation and bulge classifications. Performance metrics for correctly classifying LDD such as accuracy, sensitivity, specificity, area under the curve (AUC) and F1 were recorded, with the primary aim of the analysis to identify if one algorithm consistently outperformed others. Authors of studies who did not report accuracy or variance statistics were contacted by email for these details. Pan et al. (2021) employed an unconventional accuracy definition, incompatible with standard metrics reported by other articles. These authors did not respond to our request for standard accuracy measurements and this study was omitted from the meta-analysis [44]. Zheng and colleagues developed a DL algorithm for segmentation with additional disc measurements to diagnose LDD without ML so this study was excluded from the meta-analysis [45].

MRI acquisition parameters recorded included Tesla (T) strength and plane (axial/sagittal/transverse). MRI sequences T1W and T2W were recorded, quantitative sequences such as T1rho, T2 mapping, DIXON, spectral fat suppression were not. Standard deviation (SD) and 95% confidence intervals (CI) of performance metrics were extracted. When CI was not reported,* Z* scores (1.96) were used to transform SD to CI. In studies reporting train-test split validation without variability, SD was inferred using the variability mean of other included studies.

Sensitivity and specificity bivariate mixed effects regression was performed on studies reporting both measures (Table 2) [46]. Subsequently, a multivariate mixed effects regression of accuracy, sensitivity, specificity, AUC and F1 was performed for studies included in the meta-analysis (Table 3). Regression was fitted using rma.mv function from R package Metafor (version 3.4–0). Validity of the regression was assessed using the restricted log-likelihood plots. Logit transformation was applied to both analyses. Algorithm, LDD classification, data augmentation and internal/external validation and scaled year of publication were used as predictors. Structure of the random effects correlation between measures of each study was defined as unstructured as described before [46]. ANOVA (Wald tests) were used to group categorical variables. Post-hoc Tukey tests for significant pairwise comparisons following ANOVA were run with false discovery rate (FDR)-adjusted *p*-values. Python (3.9.12) software was used for the analysis (scipy 1.8.0, statsmodels 0.13.2). Sensitivity and specificity bivariate mixed effects regression was performed on studies reporting both measures (Table 2) [46]. Subsequently, a multivariate mixed effects regression of accuracy, sensitivity, specificity, AUC and F1 was performed for studies included in the meta-analysis (Table 3). Regression was fitted using rma.mv function from R package Metafor (version 3.4–0). Validity of the regression was assessed using the restricted log-likelihood plots. Logit transformation was applied to both analyses. Algorithm, LDD classification, data augmentation and internal/external validation were used as predictors. Structure of the random effects correlation between measures of each study was defined as unstructured as described before [46]. ANOVA (Wald tests) were used to group categorical variables. Post-hoc Tukey tests for significant pairwise comparisons following ANOVA were run with FDR-adjusted* p*-values. Python (3.9.12) software was used for the analysis Python (3.9.12) software was used for the analysis (scipy 1.8.0, statsmodels 0.13.2).

**Table 2:** Bivariate meta-regression results using sensitivity and specificity measurements

Variable	Estimate	SE	Estimate 95% CI	*Z*-value	*P*-value
Sensitivity	4.239	7.074	(− 9.625, 18.104)	0.599	0.549
Specificity	4.946	7.074	(− 8.919, 18.812)	0.699	0.484
Year of publication	1.025	6.769	(− 12.242, 14.292)	0.151	0.88
External validation	−4.169	2.745	(− 9.549, 1.21)	−1.519	0.129
Data augmentation	0.547	0.097	(0.357, 0.738)	5.626	** < .0001**
Phenotype classification#	–	–	–	–	**0.027**
Algorithm#	–	–	–	–	0.668

Table showing studies using data augmentation have higher performance than studies that did not use data augmentation. Table also shows differences in performance between different classifications of disc pathology phenotypes performance. Subsequent pairwise Tukey post-hoc analysis presented in Supplementary Table 8. # indicates multiclass variables have been summarized using Wald test ANOVA

Significance threshold at 0.05

**Table 3:** Multivariate meta-regression results using all performance measurements

Variable	Estimate	Estimate 95% CI	SE	*Z*-value	*P*-value
Year of publication	− 1.292	(− 6.126, 3.542)	2.466	− 0.524	0.601
External validation	− 2.359	(− 3.535, − 1.183)	0.6	− 3.931	** < .0001**
Data augmentation	0.668	(0.562, 0.776)	0.055	12.251	** < .0001**
Phenotype classification#	–	–	–	–	0.789
Algorithm#	–	–	–	–	0.824

Table showing studies using data augmentation have higher performance than studies that did not use data augmentation. Table also shows that external validation studies have lower performance when compared to developmental studies. # indicates multiclass variables have been summarized using Wald test ANOVA

Significance threshold at 0.05

## Results

### Types of studies

Of the 27 studies included, 22 were aimed at algorithm development, 2/27 reported development and external validation [45, 47] and 3/27 solely focused on external validation [48–50]. Most studies (24/27) used retrospective, pre-existing datasets from hospital or university collections; however, 4/27 studies prospectively examined patient scans [51–54]. Two distinct themes emerged from included articles: several had a clear focus on the underlying algorithm development and were written from a technical perspective while others, including the external validation studies, were written by and for a clinical audience. In three cases author groups published two different studies which used the same dataset [51, 55–59].

### Magnetic resonance imaging specifications

Most studies used sagittal plane MRI (21/27), but one study examined disc herniation and two studies examined disc bulge using the axial plane, while 3/27 studies used both planes. All studies reported using T2W sequences and 7/27 of them additionally used T1W sequences. From studies reporting MR field strength (22/27), most used images acquired on a 1.5 Tesla (T) scanner, some in combination with 3 T scanners. One study used a 0.4 T [52] while 3/27 studies did not report the magnetic field strength. Due to inconsistencies of MRI sequences and planes, along with several studies failing to report MR scanner field strength, MRI acquisition parameters were not included as a predictors or variables in the meta-regression. Lewandrowski et al. (2020) used T2 fast spin echo in their software that graded herniation to generate radiology reports [60].

### LDD classifications

14/27 studies used standard disease classifications including 6/27 investigating Pfirrmann grading and 3/27 MC, with one study examining both [50]. One study used numerical grading for herniation and 2/27 used descriptive grading for LDD while the remaining studies gave binary classifications for disc herniation, degeneration, or bulge. Most studies (14/27) used a single radiologist’s grading to establish ground truth. 5/27 studies used two raters, 2/27 studies used three raters and 1/27 study used four to validate ground truth. 3/27 studies did not report how MRIs were rated and 2/27 relied upon previous ratings (from medical reports). Of the studies using more than one human rater to establish ground truth, *k*-values for inter-rater agreement ranged from 0.54 [61] to 0.93 [47].

### Performance metrics and algorithms

Studies used either tenfold [51, 53, 55–57, 61, 62] five-fold [54, 59, 63–66] or random sample split validation [47, 51, 52, 55, 56, 60, 67–70]. The bivariate model of 14 studies showed differences in performances between types of classifications. Specifically, studies examining herniation had higher performance metrics than those examining disc bulge. In the bivariate model, external validation studies performed on a par with developmental studies and studies using data augmentation showed superior performance (Table 2). In the multivariate analysis of 25 studies, external validation papers did not perform as well as development studies (Table 3). Studies using data augmentation had higher performance metrics than others. However, Table 4 shows this effect is lost when studies using data augmentation are compared only to studies using large sample sizes.

**Table 4:** Multivariate meta-regression results comparing data augmented datasets with large datasets

Variable	Estimate	Estimate 95% CI	SE	*Z*-value	*P*-value
Year of publication	1.031	(−1.293, 3.354)	1.186	0.87	0.385
External validation	−0.724	(−1.047, −0.401)	0.165	−4.396	** < .0001**
Data augmentation	−0.113	(−0.581, 0.355)	0.239	−0.474	0.635
Phenotype classification#	–	–	–	–	0.043*
Algorithm#	–	–	–	–	0.92

Multivariate meta-regression only including large studies of 500 participants and over with studies that used data augmentation, showing the same performance strength between both types of studies. Table also show ANOVA difference between phenotype classification groups, however subsequent Tukey pairwise adjusted* p*-values were not significant for any comparisons (not shown)

Significance threshold at 0.05

**Fig. 4:**
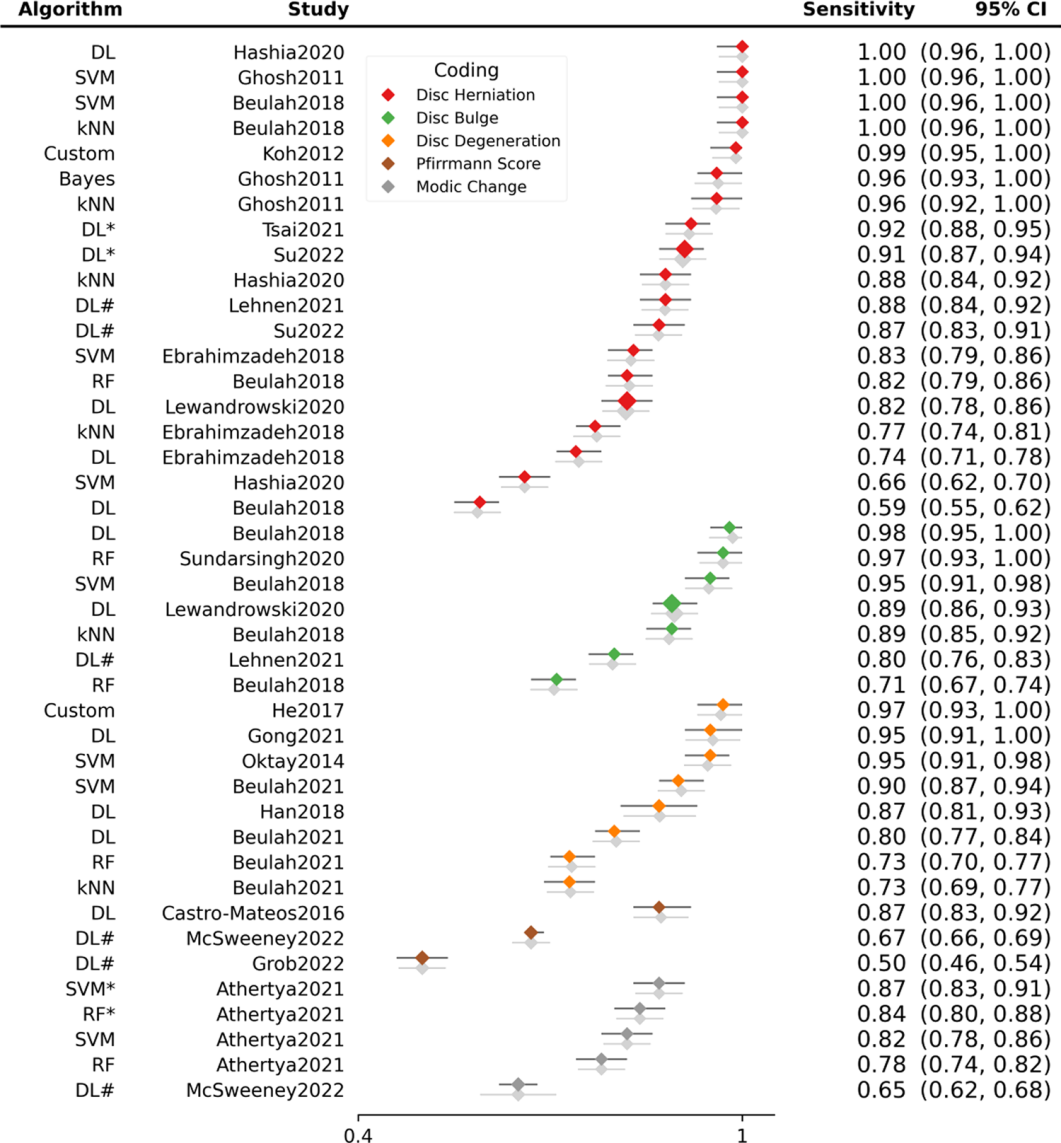
Forest plot of sensitivity. Forest plots depicting sensitivity of all algorithms examined in included studies. Grey shadow lines correspond to the DerSimonian and Laird adjusted variation. Reference marker sizes correspond to participants numbers of each study. * indicates algorithm performance with data augmentation, # indicates external validation studies. Confidence interval (CI), deep learning (DL), k nearest neighbor (kNN), random forest (RF), support vector machine (SVM)

**Fig. 5:**
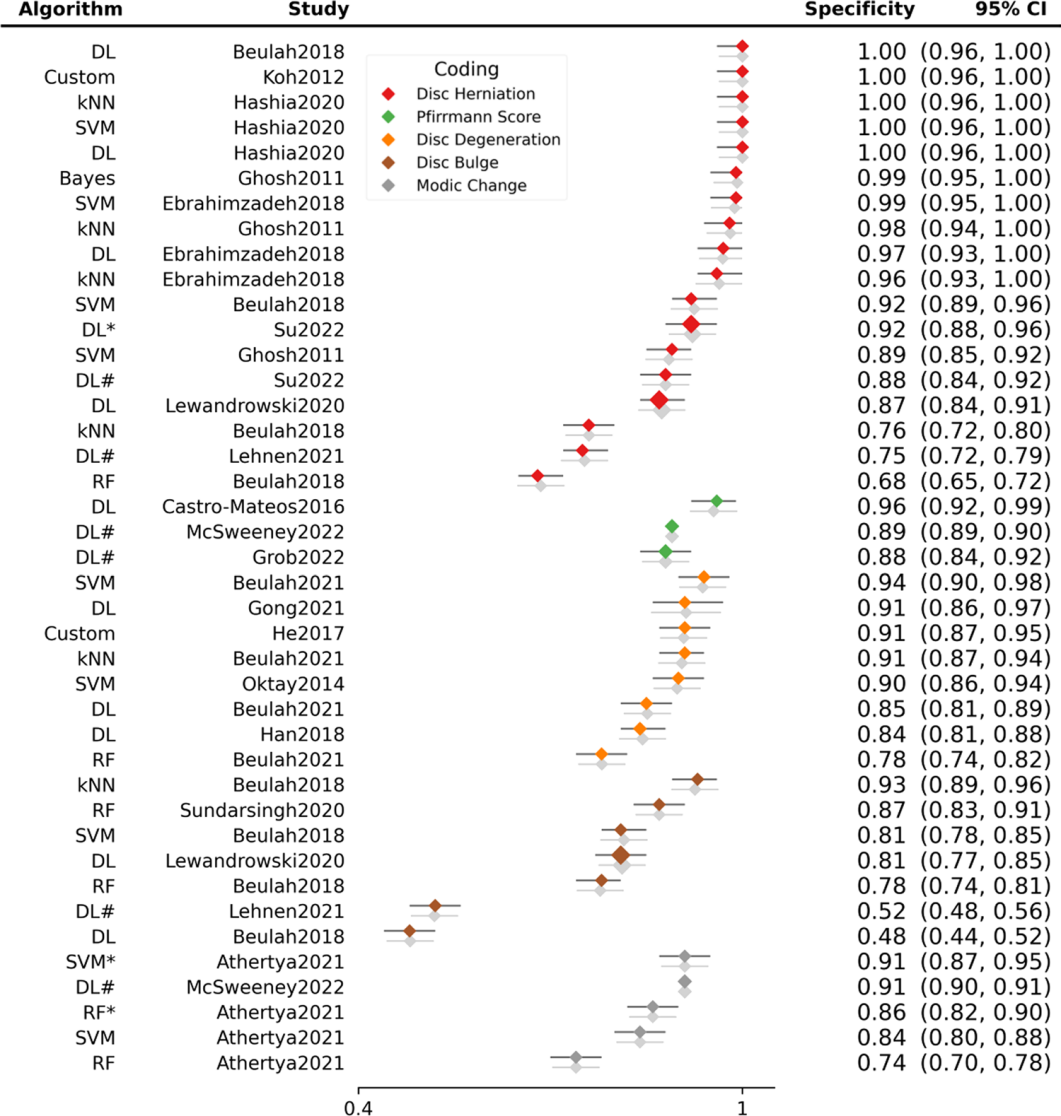
Forest plot of specificity. Forest plots depicting specificity of all algorithms examined in included studies. Grey shadow lines correspond to the DerSimonian and Laird adjusted variation. Reference marker sizes correspond to participants numbers of each study. * indicates algorithm performance with data augmentation, # indicates external validation studies. Confidence interval (CI), deep learning (DL), k nearest neighbor (kNN), random forest (RF), support vector machine (SVM)

**Fig. 6:**
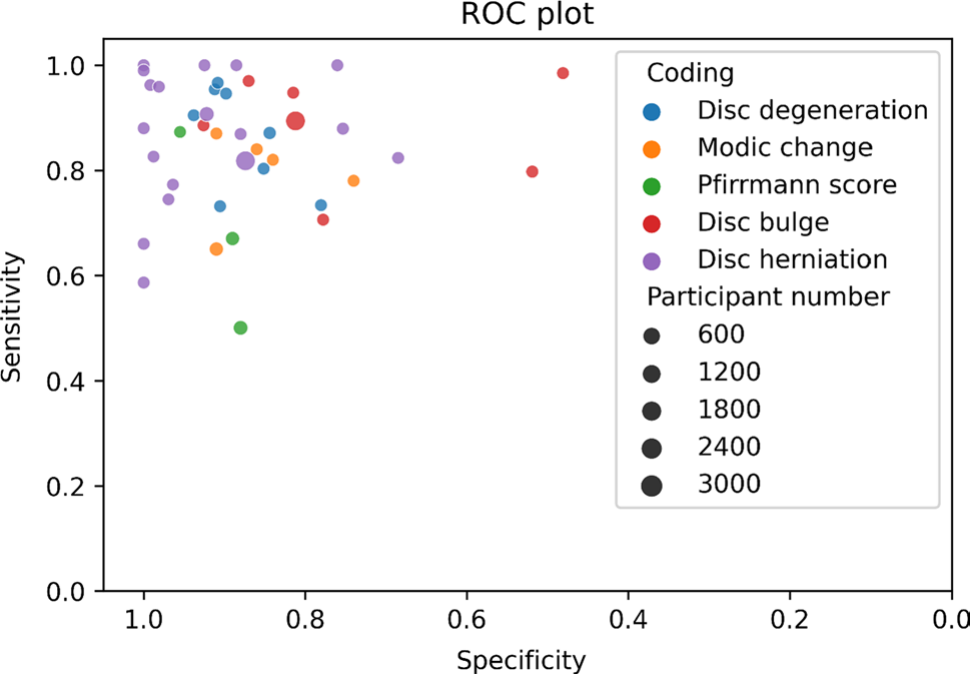
ROC plot. Receiver-operator characteristic plot of sensitivities and specificities of the published studies by coding and sample size

Sensitivity (Fig. 4) and specificity (Fig. 5) forest plots were produced. Performance receiver operating curve (ROC) is shown in Fig. 6. Forest plots depicting accuracy (S4), AUC (S5), F-1 (S6) and precision (S7) were made. These plots show the extreme heterogeneity between the included studies.

Sixteen studies with image datasets used DL algorithms, DL studies generally used large datasets, the average number of DL disc images was 4,211 (min 169, max 17,800), whereas non-DL mean disc images were 613 (min 93, max 2,500). DL models averaged an accuracy of 87.0% (SD 7.0%), specificity of 90.4% (SD 6.3%) and sensitivity 88.2% (SD 7.2%). 8/25 studies compared performance of several algorithms, including DL, SVM, kNN, NB and RF. Other studies used nonspecific algorithms which were listed as custom models (2/25).

### Risk of bias and quality assessment

There were large amounts of missing information: many studies did not report ethical approval, participant consent or waiving of consent for retrospectively designed studies. Basic participant details were missing; participants’ mean age, or recruitment site were often not reported. The PROBAST tool, completed and agreed by RC and IGS ranked 7/27 studies as *low*, 16/27 *unclear* and 4/27 *high* risk of bias (S10). For many studies performance measures and CI information were missing. We contacted authors of 11/27 studies asking for the variance of reported statistics; of these two responded—but only one provided the requested information. Only 2/27 studies provided statements of data availability [45, 47]—this is now standard in fields like genetic epidemiology—and just one study provided a link to their algorithm code [45]. PROBAST judgments of the applicability of included studies was generally poor—however most included studies were developmental rather than models that could immediately translate to useable clinical or research tools. For several questions assessing study quality and applicability many papers did not include any or appropriate detail. Information was mainly missing for the following four questions, prohibiting judgments quality and applicability:

3.1 Was the outcome determined appropriately?
4.4 Were participants with missing data handled appropriately?
4.3 Were all enrolled participants included in the analysis?
4.7 Were relevant model performance measures evaluated appropriately?

## Discussion

Most studies included in this systematic review used DL models for LDD, bulge or herniation classification or grading by Pfirrmann or MC criteria. DL models, made up of complex layered input networks, might be expected to surpass other algorithms for reading spine MRI. This expectation was echoed by the choice of many authors of studies in this review to utilize DL models. However, our meta-analysis results do not show differences in DL performance compared to other ML approaches. DL algorithms tend to have a high false positive error rate and are difficult to calibrate [71]. Development and validation of SpineNet predominated DL studies. While used for research purposes, this model may be the most suitable candidate to develop for clinical practice. The software can detect six spine abnormalities including MC with ~ 87% accuracy, [72] Pfirrmann grade, disc narrowing, spondylolisthesis and central canal stenosis [73]. External validation of SpineNet demonstrated a balanced accuracy of 76% for Pfirrmann and 78% for MC classification in a large population dataset [50] and class average accuracy of 55% for Pfirrmann grade classification [48]. An external validation of an open-source version of SpineNet (V2) [73] is currently under review.

Supervised models predominate medical research; however performance improvements may come from incorporating other training techniques such as semi-supervised learning which labels a small portion of a large training dataset. Subsequent unlabelled data can improve classification results. Lewandroski and colleagues (2020) successfully combined supervised, semi-supervised and unsupervised training of a natural language processing (NLP) algorithm to generate radiologist reports. A 3D model was fitted to MRIs (axial, sagittal and transverse) in a large dataset, with high performance metrics reported for separate DL models that graded disc bulge and herniation and stenosis. Detailed radiological reports with only trivial differences from human reports were generated [60]. This innovative study design demonstrates the effective use of semi- and unsupervised learning with NLP, an addition which could be adopted to DL reading of MRIs.

Two DL studies used extended Pfirrmann grading systems—which allow for uncertainties between exact categories [61], and reported high agreement with ground truth [45, 61]. Niemeyer et al., (2021) compared analyses that treated Pfirrmann grades as categorical, numerical and continuous variables, and found a linear regression of a continuous variable had higher numbers of slight errors but gave best results overall, with fewer large deviations from ground truth [61]. The extended scale had good correlation with disc hydration and other markers of degeneration [45]. Zheng et al., (2022) went on to externally validate their model in a small dataset and have sought intellectual property rights for the software, proposing their model can easily integrate into existing MRI systems [45].

Pan et al. (2021) successfully integrated three CNN models for location, definition and classification of discs and used ResNet modification to reduce overfitting [44]. Gao et al., (2021) reported similarly high accuracy to Pan et al. (2021), adding a regularization method to their two CNN models to enhance separation of differential features. Classification according to Pfirrmann categories is a challenge for DL and CNN, with many samples “between grades”—training samples are often at the margins of a grade, so can easily be misclassified in testing, reducing model performance [53]. A more complex architecture including a memory component to reduce overfitting and include information about neighboring tissue was used by Han et al.’s (2018) CNN model, resulting in very high performance. This study demonstrates architectural modifications can incorporate information from a range of spine tissues captured in MRI rather than limiting to a single structure [63]. In a similar fashion Gong et al. (2021) used several features of surrounding spine tissues in axial MRIs, positing axial images may better capture multiple structures [54].

Castro-Mateos et al.’s. (2016) small, prospective study compared four different algorithm classifiers and found NN-2 performed best. Authors note an effective system must pick up on features not detected by human radiologists; while not included in their design, this implies that a component of unsupervised training may have benefitted their model [52]. While these CNN models perform well, a specific constraint to this study and an over-arching limitation of the review is the incompatibility of Pfirrmann grading with LDD progression which presents a challenge to algorithm performance.

Linear approaches commonly used include support vector machines (SVM) that are ‘noise-tolerant’ classifiers with reduced propensity to overfitting, however these algorithms tend not to handle complex or extensive data [71]. SVM are typically used for classification problems, and rely on linearly separating data, based on pattern differences. ‘Support vectors’ are datapoints on the periphery of a category—thus they support the plane of separation or decision boundary. SVM are most successful in datasets where large margins between categories exist. As described, the Pfirrmann grading system may be more precise when used as a continuous than categorical measure, which more accurately reflects the progression of degenerating discs [45, 61]. Stand-out SVM results include those from Oktay and colleagues (2014) which achieved high accuracy identifying disc herniation by extracting several image features (intensity, texture, whole shape, context) and focusing on the difference image created by comparing T1W and T2W MRIs [74]. They state that using the difference image helps to disregard artifacts of either T1W or T2W images [74]. SVM is computationally inexpensive, therefore attractive for model development. For these models to perform well, images must be simplified with pre-processing techniques such as grey scale reduction [51, 57, 70, 74], window cropping [56], and thresholding [55]. Conceptually SVM appears less capable of handling the complexities of identifying disc pathology in MRIs than DL models.

Herniation classification was tested with kNN, SVM, NB and two types of dimensionality reduction by Ghosh et al. (2011) who found concatenating different texture, shape and intensity features improved performance [64]. Using a RF classifier to diagnose disc budge and desiccation, Sundarsingh and Kesavan (2020) also found combining one texture and one shape feature improved accuracy [68]. These small studies collectively report increased success with the incorporation of multiple features.

It is common to use small datasets for algorithm development, big data are available but often require extensive cleaning and preparation [75]. The use of data augmentation is prevalent among AI-developers, the creation of additional training data can improve validation performance [76]. Six studies in our review used varying extents of augmentation. Athertya and colleagues identified MC1 and MC2 cases in images from 100 participants [55, 56]. Ten MC1 cases were augmented to 160 using synthetic minority over sampling technique and MC2 cases were doubled from 88 to 176 [55, 56]. It is difficult to acquire ‘case’ scans for training, however a curated dataset of original MRIs may provide a better training tool than one so aggressively augmented [77, 78]. Other studies have also employed extreme augmentation, for example Tsai and colleagues (2021) used rotation and contrast and brightness augmentation ran several trials augmenting between 50 and 550 images, with the model maintaining high accuracy [67]. Niemeyer and colleagues (2021) used elaborate pre-processing [61], not undertaken in usual care which does reduce applicability, however this model outperformed most others in the review, and it will be interesting to see if training with such manicured data translates to good performance in any subsequent external validation studies. Jamaludin and colleagues (2016) produced a texture feature extraction guided algorithm to assess MC from images focused on features of vertebral endplates. They used data augmentation, in a similar, but less extreme fashion to Niemeyer et al. (2021), in both training and testing datasets posited to better reflect real-world circumstances [66]. Su et al. (2022) also employed a lighter touch, randomly cropping and rotating training images to enhance real-world applicability of their model [47]. MRI images may be less amenable to augmentation than non-medical, natural image classification tasks—augmenting scans may distort MRI datasets, creating unrealistic images [78]. Augmentation specifically designed for fMRI showed a 26% algorithm performance improvement over more modest traditional augmentation results [78, 79].

AI promises exciting developments such as the prediction of patient response to treatment—and differentiating patients to optimal treatments. The onus to publish novel findings is not limited to computer science. The performance of any algorithm purported to assist medicine needs to be well- and repeatedly validated, however we only found three replication studies [48–50]. CoLumbo software appears to have been marketed to radiology departments based on one published external validation study [49]. In this study, authors note the software could benefit from future improvement, yet it is unclear if such improvements have been undertaken. We contacted the company for publications validating the software and received documentation reporting a trial in three hospitals conducted to receive EU Medical Devices Directive approval (available from CoLumbo marketing). External validation of successful algorithms remains an essential part of real-world applicability. Another recent publication testing a “widely available algorithm” used for LDD grading reported it failed significantly on unseen data [79]. The algorithm being tested was not made public and their work has not been included in this review, but findings present a cautionary tale against blind acceptance of model outputs [79]. It also demonstrates the need for new ground truth labels, repeated validation studies and close surveillance of software performance integrity.

## Strengths and limitations

This systematic review has some limitations. Firstly, several studies had very small sample sizes. Nikravan, Ebrahimzadeh and colleagues developed binary herniation classifiers. Using SVM and NN [59] followed by a kNN algorithm, which used grey thresholding intensity features to identify disc rupture and leaking annulus pulposus [58]. Good performance was reported in both studies with the same 30 participants, but only limited generalizability from samples of this size may be inferred [52, 58, 59, 62, 64].

Included studies had either a strong computer science approach or a clinical focus. A second limitation was that computer science-focused reports often omitted participant details. Reported performance metrics differed between studies and many lacked variance statistics, posing challenges to meta-analysis; frequently ethical committee statements, basic participant details, missing data contingencies, basic model variance statistics or reference to disc degeneration grading systems were missing—and none of the development studies reported a contingency table. Incomplete reporting has been documented by other reviewers, who have called for “international standards for study protocols and reporting that recognize challenges” inherent to DL and ML [80]. A recent large systematic review and meta-analysis of ML for medical diagnosis found that while speciality DL algorithms were *generally* clinically accurate, there was unacceptably high variation in reporting of metrics, methodology and reference standards [81]. Parsimonious reporting is an unnecessary waste in biomedical research [82] and robust clinical peer review is lacking, with a dearth of randomized trials, prospective or external validation studies; estimated as low as 6% of all published AI studies [83, 84].

The third significant limitation was that studies performed poorly in the PROBAST risk of bias and quality assessment. This is in part due to the lack of availability of an AI-specific assessment tool. We and others are frustrated by the lack of an AI-specific tool and anticipate the Delphi group’s publication of tools like TRIPOD-AI [41, 80].

## Conclusion

MRI reading may be most suited to DL processing, with the presentation of greater quantities and more varied data. Semi- and unsupervised components will improve the chance of detecting patterns that currently elude human radiologists. Successful models will likely benefit from using greater numbers of features and the incorporation of information from tissue surrounding the disc. The use of multiple MRI planes and all clinical sequencies, along with incorporating the T1W and T2W difference image, may provide richer data for ML algorithms to process. Employing continuous grading classifications, more sympathetic to the progression of LDD may also be useful.

We encourage the use and sharing of large datasets for developing and validating models. While data augmentation may seem an attractive bypass, susceptibility to model overfitting threatens practical performance. Progression of this field is hampered by lack of external validation studies, although such work is the backbone of any robust model in any field.

The current scientific and reporting quality of ML studies to identify LDD is overall insufficient, and none have been reliably implemented as a clinical decision-making tool. Widely acceptable methodological and reporting guidelines for ML in LDD research are warranted yet remain unavailable. We attempted to pursue missing data to improve the strength of our conclusions, with limited success. Future research should aim to bridge the gap between biomedical engineering literature and clinical value of the software. This review highlights the need to move beyond simply matching radiologists' interpretation to extracting quantitative LDD representations that effectively utilize the full complexity of data contained in spine MRI sequencing.

## Supplementary Information

Below is the link to the electronic supplementary material.

Supplementary file1 (DOCX 13 KB)

Supplementary file2 (DOCX 13 KB)

Supplementary file3 (PDF 76 KB)

Supplementary file4 (DOC 32 KB)

Supplementary file5 (DOCX 24 KB)

Supplementary file6 (DOCX 352 KB)

Supplementary file7 (DOCX 132 KB)

Supplementary file8 (DOCX 151 KB)

Supplementary file9 (DOCX 149 KB)

Supplementary file10 (DOCX 17 KB)

Supplementary file11 (DOCX 19 KB)

Supplementary file12 (DOCX 12 KB)

Supplementary file13 (DOCX 34 KB)

## Data Availability

Supplementary documents provided detailing systematic search strategy.
