# Supplementary material for: Are current machine learning applications comparable to radiologist classification of degenerate and herniated discs and Modic change? A systematic review and meta-analysis
Source: Eur Spine J. 2023 May 8;32(11):3764–87. doi: 10.1007/s00586-023-07718-0 (PMC10164619; doi:10.1007/s00586-023-07718-0)
Supplement: Supplementary file 1 — Supplementary file1 (DOCX 13 KB) [file 586_2023_7718_MOESM1_ESM.docx]

Are current machine learning applications analogous to radiologist classification of degenerate discs? A systematic review and meta-analysis.

Search strategy:

1350 articles were extracted, and 182 duplicates removed. Title and abstract searching identified 173 candidate papers; of which 18 were included in the review. Reference list searches of included articles were conducted, with three additional articles identified for inclusion.

Prior to the completion of the manuscript first draft, the same search was re-conducted on 17.04.22 to identify any recent publications and four more articles including one abstract were identified. Two articles were identified by data base search alerts. Finally, 27 studies were identified as eligible and included in the review (Table 1). A decision to halt the inclusion of more recent publications was made on 25.07.22, as the first manuscript draft was finalised.

Databases:

Cihal, Embase, PubMed, Web of Science (includes Medline),

*Search strings*

Concept1

endplate OR intervertebral disc OR disc degeneration OR Modic change OR Schmorls nodes OR myelopathy OR spondylosis OR Pfirrmann

Concept 2

MRI OR magnetic resonance imaging OR radiology OR 3D medical images OR 3D CT OR imaging

Concept 3

Artificial intelligence OR machine learning OR computer learning OR reinforcement learning OR supervised learning OR unsupervised learning OR computer vision OR deep learning OR neural networks OR NN OR artificial neural networks OR ANN OR texture analysis

Concept 4

Prediction OR prognosis OR outcome OR patient outcome OR diagnosis OR identification OR detection OR true positive

*MeSH terms*

Imaging

Disc degeneration/disc pathology

Machine learning

Prognosis/diagnosis

*Search limits*

Human, Journal article
