# Supplementary material for: Are current machine learning applications comparable to radiologist classification of degenerate and herniated discs and Modic change? A systematic review and meta-analysis
Source: Eur Spine J. 2023 May 8;32(11):3764–87. doi: 10.1007/s00586-023-07718-0 (PMC10164619; doi:10.1007/s00586-023-07718-0)
Supplement: Supplementary file 2 — Supplementary file2 (DOCX 13 KB) [file 586_2023_7718_MOESM2_ESM.docx]

Supplementary 2.

Grey literature search details

A grey literature search was conducted which involved search mesh terms disc degeneration, imaging and machine learning on grey databases and contacting recognised academics and researchers for unpublished relevant articles. Embase and Web of Science search conference proceedings and dissertations so it was decided these aspects of grey literature searching were accounted for in our primary search.

MeSH terms

Disc degeneration, Imaging, Machine learning

Grey literature searches were performed, with mesh terms. Trial registry searches are reported below, where the term ‘disc degeneration’ failed to reveal any results the term ‘back pain’ was used. No trials were found that were relevant to the current systematic review.

NIHR search ‘back pain’ 24.04.2022 https://bepartofresearch.nihr.ac.uk/results/search-results?query=Back%20pain&location=

Clinical trials.gov ‘back pain’ and ‘machine learning’ revealed one interesting study FUSE-ML which aimed to use ML to predict an optimal surgery decision for back pain patients. FUSE-ML algorithm did not use MRI data and hence the study not included https://clinicaltrials.gov/ct2/show/record/NCT05161130?term=machine+learning&cond=Back+Pain&draw=2&rank=1

The ISRCTN registry was searched using the term ‘disc degeneration’ https://www.isrctn.com/search?pageSize=100&sort=&page=1&q=disc+degeneration&filters=&searchType=advanced-search

Grey literature databases National Grey Literature Collection, Trip and OpenAIRE, using the terms Disc degeneration AND Imaging AND Machine learning and found 15, 71 and 5 hits respectively. Google Scholar using the terms Intervertebral disc degeneration AND Imaging AND Machine learning with a 2022 date limit and 2,050 articles were retrieved on 27.04.22. The first 200 Google Scholar articles were checked, however none of the grey literature databases revealed additional suitable articles.
