# Supplementary material for: Are current machine learning applications comparable to radiologist classification of degenerate and herniated discs and Modic change? A systematic review and meta-analysis
Source: Eur Spine J. 2023 May 8;32(11):3764–87. doi: 10.1007/s00586-023-07718-0 (PMC10164619; doi:10.1007/s00586-023-07718-0)
Supplement: Supplementary file 3 — Supplementary file3 (PDF 76 KB) [file 586_2023_7718_MOESM3_ESM.pdf]

Print Search History

web.p.ebscohost.com/ehost/searchhistory/PrintSearchHistory

Accessibility Information and Tips

Thursday, April 21, 2022 10:08:22 AM

EBSCOhost

#	Query	Limiters/Expanders	Last Run Via	Results

S4	S1 AND S2 AND S3	Expanders - Apply equivalent subjects<br>Search modes - Boolean/Phrase	Interface - EBSCOhost Research Databases<br>Search Screen - Advanced Search<br>Database - Academic Search Complete;AHFS Consumer Medication Information;APA PsycInfo;Art & Architecture Complete;Biography Reference Center;Biography Reference eBook Collection;Business Source Complete;CINAHL Complete;CINAHL Plus with Full Text;Cochrane Central Register of Controlled Trials;Cochrane Clinical Answers;Cochrane Database of Systematic Reviews;Cochrane Methodology Register;Current Biography Illustrated (H.W. Wilson);eBook Academic Collection (EBSCOhost);eBook Business Collection (EBSCOhost);eBook Collection (EBSCOhost);eBook Open Access (OA) Collection (EBSCOhost);Entrepreneurial Studies Source;Environment Complete;ERIC;GreenFILE;Health Source - Consumer Edition;Health Source: Nursing/Academic Edition;Library, Information Science & Technology Abstracts with Full Text;Literary Reference Center Plus;Literary Reference eBook Collection;MasterFILE Premier;MasterFILE Reference eBook Collection;Mental Measurements Yearbook with Tests in Print;Military & Government Collection;Newspaper Source Plus;Newswires;OmniFile Full Text Mega (H.W. Wilson);Points of View Reference Center;Regional Business News;Teacher Reference Center;Web News	303

S3	artificial intelligence or machine learning or computer learning or reinforcement learning or supervised learning or unsupervised learning or computer vision or deep learning or neural network or NN or artificial neural network or SVM or random forest or CNN or Naïve Bayes or KNN or Decision Tree	Expanders - Apply equivalent subjects<br>Search modes - Boolean/Phrase	Interface - EBSCOhost Research Databases<br>Search Screen - Advanced Search<br>Database - Academic Search Complete;AHFS Consumer Medication Information;APA PsycInfo;Art & Architecture Complete;Biography Reference Center;Biography Reference eBook Collection;Business Source Complete;CINAHL Complete;CINAHL Plus with Full Text;Cochrane Central Register of Controlled Trials;Cochrane Clinical Answers;Cochrane Database of Systematic Reviews;Cochrane Methodology Register;Current Biography Illustrated (H.W. Wilson);eBook Academic Collection (EBSCOhost);eBook Business Collection (EBSCOhost);eBook Collection (EBSCOhost);eBook Open Access (OA) Collection (EBSCOhost);Entrepreneurial Studies Source;Environment Complete;ERIC;GreenFILE;Health Source - Consumer Edition;Health Source: Nursing/Academic Edition;Library, Information Science & Technology Abstracts with Full Text;Literary Reference Center Plus;Literary Reference eBook Collection;MasterFILE Premier;MasterFILE Reference eBook Collection;Mental Measurements Yearbook with Tests in Print;Military & Government Collection;Newspaper Source Plus;Newswires;OmniFile Full Text Mega (H.W. Wilson);Points of View Reference Center;Regional Business News;Teacher Reference Center;Web News	1,632,973

S2	intervertebral disk or intervertebral or endplate or intervertebral disc or disc degeneration or Modic change or Schmorls nodes or myelopathy or spondylosis or Pfirrmann or spinal stenosis	Expanders - Apply equivalent subjects<br>Search modes - Boolean/Phrase	Interface - EBSCOhost Research Databases<br>Search Screen - Advanced Search<br>Database - Academic Search Complete;AHFS Consumer Medication Information;APA PsycInfo;Art & Architecture Complete;Biography Reference Center;Biography Reference eBook Collection;Business Source Complete;CINAHL Complete;CINAHL Plus with Full Text;Cochrane Central Register of Controlled Trials;Cochrane Clinical Answers;Cochrane Database of Systematic Reviews;Cochrane Methodology Register;Current Biography Illustrated (H.W. Wilson);eBook Academic Collection (EBSCOhost);eBook Business Collection (EBSCOhost);eBook Collection (EBSCOhost);eBook Open Access (OA) Collection (EBSCOhost);Entrepreneurial Studies Source;Environment Complete;ERIC;GreenFILE;Health Source - Consumer Edition;Health Source: Nursing/Academic Edition;Library, Information Science & Technology Abstracts with Full Text;Literary Reference Center Plus;Literary Reference eBook Collection;MasterFILE Premier;MasterFILE Reference eBook Collection;Mental Measurements Yearbook with Tests in Print;Military & Government Collection;Newspaper Source Plus;Newswires;OmniFile Full Text Mega (H.W. Wilson);Points of View Reference Center;Regional Business News;Teacher Reference Center;Web News	110,862

S1	MRI or magnetic resonance or MR imaging or radiology or medical images or "CT" orcomputed tomography or imaging or X-ray	Expanders - Apply equivalent subjects<br>Search modes - Boolean/Phrase	Interface - EBSCOhost Research Databases<br>Search Screen - Advanced Search<br>Database - Academic Search Complete;AHFS Consumer Medication Information;APA PsycInfo;Art & Architecture Complete;Biography Reference Center;Biography Reference eBook Collection;Business Source Complete;CINAHL Complete;CINAHL Plus with Full Text;Cochrane Central Register of Controlled Trials;Cochrane Clinical Answers;Cochrane Database of Systematic Reviews;Cochrane Methodology Register;Current Biography Illustrated (H.W. Wilson);eBook Academic Collection (EBSCOhost);eBook Business Collection (EBSCOhost);eBook Collection (EBSCOhost);eBook Open Access (OA) Collection (EBSCOhost);Entrepreneurial Studies Source;Environment Complete;ERIC;GreenFILE;Health Source - Consumer Edition;Health Source: Nursing/Academic Edition;Library, Information Science & Technology Abstracts with Full Text;Literary Reference Center Plus;Literary Reference eBook Collection;MasterFILE Premier;MasterFILE Reference eBook Collection;Mental Measurements Yearbook with Tests in Print;Military & Government Collection;Newspaper Source Plus;Newswires;OmniFile Full Text Mega (H.W. Wilson);Points of View Reference Center;Regional Business News;Teacher Reference Center;Web News	4,088,168
