# Supplementary material for: Are current machine learning applications comparable to radiologist classification of degenerate and herniated discs and Modic change? A systematic review and meta-analysis
Source: Eur Spine J. 2023 May 8;32(11):3764–87. doi: 10.1007/s00586-023-07718-0 (PMC10164619; doi:10.1007/s00586-023-07718-0)
Supplement: Supplementary file 4 — Supplementary file4 (DOC 32 KB) [file 586_2023_7718_MOESM4_ESM.doc]

Database: Embase Classic+Embase <1947 to 2021 June 18>

Search Strategy:

--------------------------------------------------------------------------------

1 (intervertebral disk or intervertebral or endplate or intervertebral disc or disc degeneration or Modic change or Schmorls nodes or myelopathy or spondylosis or Pfirrmann or spinal stenosis).mp. [mp=title, abstract, heading word, drug trade name, original title, device manufacturer, drug manufacturer, device trade name, keyword, floating subheading word, candidate term word] (102169)

2 (MRI or magnetic resonance or MR imaging or radiology or medical images or CT or computed tomography or imaging or X-ray).mp. [mp=title, abstract, heading word, drug trade name, original title, device manufacturer, drug manufacturer, device trade name, keyword, floating subheading word, candidate term word] (4120203)

3 (artificial intelligence or machine learning or computer learning or reinforcement learning or supervised learning or unsupervised learning or computer vision or deep learning or neural network or NN or artificial neural network or SVM or random forest or CNN or Naive Bayes or knn or Decision Tree).mp. [mp=title, abstract, heading word, drug trade name, original title, device manufacturer, drug manufacturer, device trade name, keyword, floating subheading word, candidate term word] (210461)

4 1 and 2 and 3 (146)

***************************

PUBMED Search strings 18.06.21

Search: (("endplate" OR "intervertebral disc" OR "disc degeneration" OR "Modic change" OR "Schmorls nodes" OR "myelopathy" OR "spondylosis" OR "Pfirrmann") AND ("MRI" OR "magnetic resonance imaging" OR "radiology" OR "3D medical images" OR "3D CT" OR "imag*")) AND ("Artificial intelligence" OR "machine learning" OR "computer learning" OR "reinforcement learning" OR "supervised learning" OR "unsupervised learning" OR "computer vision" OR "deep learning" OR "neural networks" OR "NN" OR "artificial neural networks" OR "ANN" OR "texture analysis")

“endplate” OR “intervertebral disc” OR “disc degeneration” OR “Modic change” OR “Schmorls nodes” OR “myelopathy” OR “spondylosis” OR “Pfirrmann”

AND

“MRI” OR “magnetic resonance imaging” OR “radiology” OR “3D medical images” OR “3D CT” OR “imag*”

AND

“Artificial intelligence” OR “machine learning” OR “computer learning” OR “reinforcement learning” OR “supervised learning” OR “unsupervised learning” OR “computer vision” OR “deep learning” OR “neural networks” OR “NN” OR “artificial neural networks” OR “ANN” OR “texture analysis”

MeSH terms

“Imag*”

“Dis? Degeneration” /disc pathology

“Machine learning”
