# Supplementary material for: Are current machine learning applications comparable to radiologist classification of degenerate and herniated discs and Modic change? A systematic review and meta-analysis
Source: Eur Spine J. 2023 May 8;32(11):3764–87. doi: 10.1007/s00586-023-07718-0 (PMC10164619; doi:10.1007/s00586-023-07718-0)
Supplement: Supplementary file 5 — Supplementary file5 (DOCX 24 KB) [file 586_2023_7718_MOESM5_ESM.docx]

|  | | | | |
| --- | --- | --- | --- | --- |
| # 5 | [**428**](https://apps.webofknowledge.com/summary.do?product=UA&doc=1&qid=18&SID=E1SEJQQxZpWaGVSHE51&search_mode=CombineSearches&update_back2search_link_param=yes) | #3 AND #2 AND #1  **Refined by:** **DOCUMENT TYPES:** ( ARTICLE )  *Databases= WOS, KJD, MEDLINE, RSCI, SCIELO Timespan=All years*  *Search language=Auto* |  |  |
|  | | | | |
| # 4 | [**504**](https://apps.webofknowledge.com/summary.do?product=UA&doc=1&qid=17&SID=E1SEJQQxZpWaGVSHE51&search_mode=CombineSearches&update_back2search_link_param=yes) | #3 AND #2 AND #1  *Databases= WOS, KJD, MEDLINE, RSCI, SCIELO Timespan=All years*  *Search language=Auto* |  |  |
|  | | | | |
| # 3 | [**7,985,352**](https://apps.webofknowledge.com/summary.do?product=UA&doc=1&qid=16&SID=E1SEJQQxZpWaGVSHE51&search_mode=AdvancedSearch&update_back2search_link_param=yes) | TS=(MRI or magnetic resonance or MR imaging or radiology or medical images or CT or computed tomography or imaging or X-ray)  *Databases= WOS, KJD, MEDLINE, RSCI, SCIELO Timespan=All years*  *Search language=Auto* |  |  |
|  | | | | |
| # 2 | [**1,885,041**](https://apps.webofknowledge.com/summary.do?product=UA&doc=1&qid=15&SID=E1SEJQQxZpWaGVSHE51&search_mode=AdvancedSearch&update_back2search_link_param=yes) | TS= (artificial intelligence or machine learning or computer learning or reinforcement learning or supervised learning or unsupervised learning or computer vision or deep learning or neural network or NN or artificial neural network or SVM or random forest or CNN or Naive Bayes or knn or Decision Tree)  *Databases= WOS, KJD, MEDLINE, RSCI, SCIELO Timespan=All years*  *Search language=Auto* |  |  |
|  | | | | |
| # 1 | [**92,293**](https://apps.webofknowledge.com/summary.do?product=UA&doc=1&qid=14&SID=E1SEJQQxZpWaGVSHE51&search_mode=AdvancedSearch&update_back2search_link_param=yes) | TS= (endplate* OR Intervertebral dis? OR disc degenerate* OR Modic change* OR IDD OR Schmorls nodes OR myelopathy OR spondylosis OR spinal stenosis OR Pfirrmann)  *Databases= WOS, KJD, MEDLINE, RSCI, SCIELO Timespan=All years*  *Search language=Auto* |  |  |

Web of Science 18.06.21
