# Supplementary material for: Are current machine learning applications comparable to radiologist classification of degenerate and herniated discs and Modic change? A systematic review and meta-analysis
Source: Eur Spine J. 2023 May 8;32(11):3764–87. doi: 10.1007/s00586-023-07718-0 (PMC10164619; doi:10.1007/s00586-023-07718-0)
Supplement: Supplementary file 7 — Supplementary file7 (DOCX 132 KB) [file 586_2023_7718_MOESM7_ESM.docx]

Supplementary Figure 5. Modic change forest plot of area under the curve


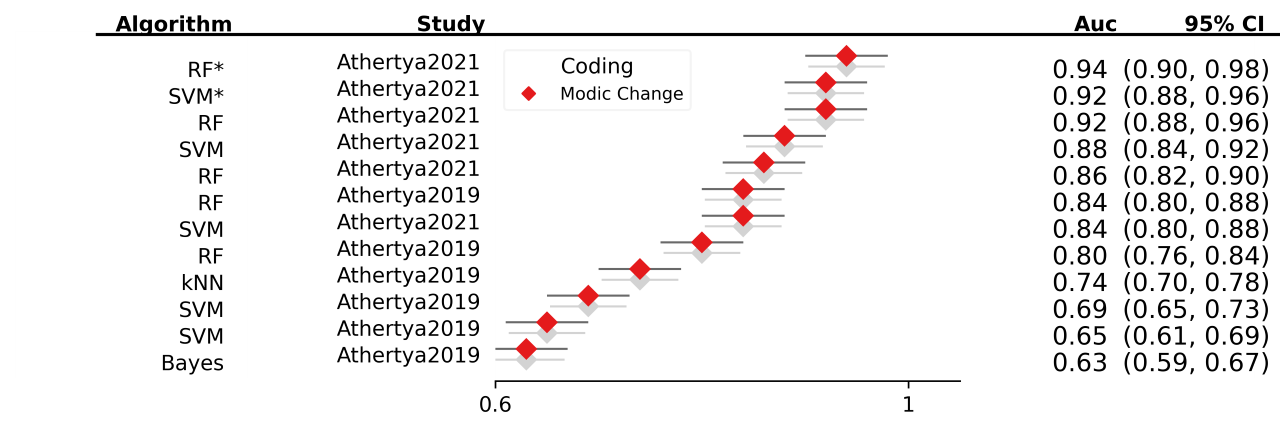


Forest plots depicting area under the curve of all algorithms examined in included studies. Grey shadow lines correspond to the DerSimonian and Laird adjusted variation. Reference marker sizes correspond to participants numbers of each study. * indicates algorithm performance with data augmentation, # indicates external validation studies.

Confidence interval (CI), k nearest neighbour (kNN), random forest (RF), support vector machine (SVM).
