# Supplementary material for: Are current machine learning applications comparable to radiologist classification of degenerate and herniated discs and Modic change? A systematic review and meta-analysis
Source: Eur Spine J. 2023 May 8;32(11):3764–87. doi: 10.1007/s00586-023-07718-0 (PMC10164619; doi:10.1007/s00586-023-07718-0)
Supplement: Supplementary file 9 — Supplementary file9 (DOCX 149 KB) [file 586_2023_7718_MOESM9_ESM.docx]

Supplementary Figure 7. Forest plot of precision scores


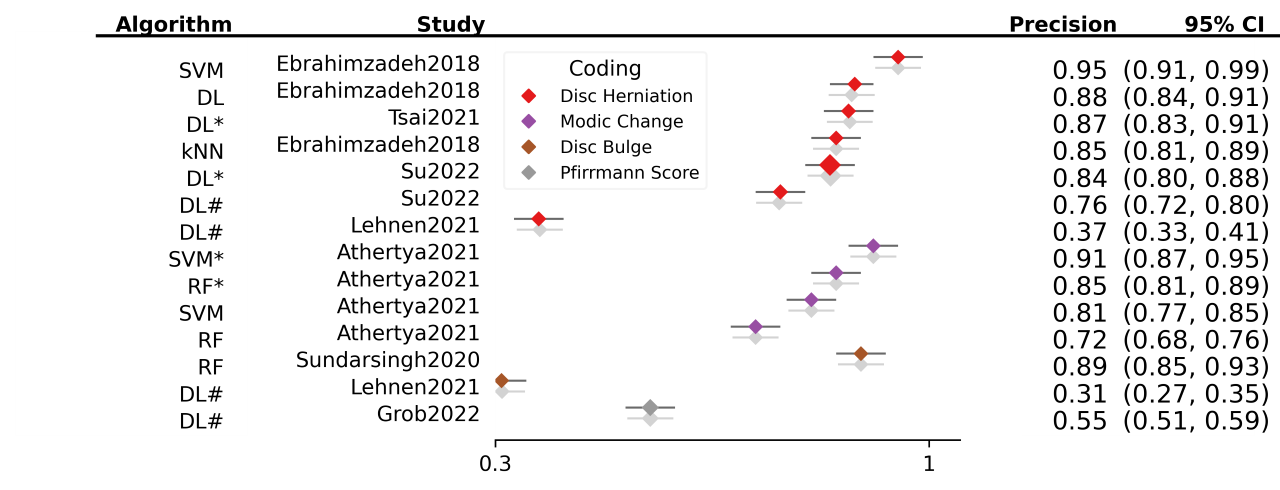


Forest plots depicting precision of all algorithms examined in included studies. Grey shadow lines correspond to the DerSimonian and Laird adjusted variation. Reference marker sizes correspond to participants numbers of each study. * indicates algorithm performance with data augmentation, # indicates external validation studies.

Confidence interval (CI), deep learning (DL), k nearest neighbour (kNN), random forest (RF), support vector machine (SVM).
