# Supplementary material for: Are current machine learning applications comparable to radiologist classification of degenerate and herniated discs and Modic change? A systematic review and meta-analysis
Source: Eur Spine J. 2023 May 8;32(11):3764–87. doi: 10.1007/s00586-023-07718-0 (PMC10164619; doi:10.1007/s00586-023-07718-0)
Supplement: Supplementary file 10 — Supplementary file10 (DOCX 17 KB) [file 586_2023_7718_MOESM10_ESM.docx]

Supplementary Table 8. Post-hoc Tukey pairwise comparisons of phenotype classifications

| **Reference** | **Comparison** | **Estimate** | **Estimate 95% CI** | **SE** | **Z-value** | **P-value** | **P-adjusted** |
| --- | --- | --- | --- | --- | --- | --- | --- |
| LDD | Bulge | -1.243 | (-9.433, 6.947) | 2.555 | -0.486 | 0.627 | 0.853 |
| MC | Bulge | -0.013 | (-10.810, 10.784) | 3.368 | -0.004 | 0.997 | 0.997 |
| Pfirrmann | Bulge | 1.207 | (-10.004, 12.418) | 3.497 | 0.345 | 0.730 | 0.853 |
| MC | LDD | 1.230 | (-9.557, 12.017) | 3.365 | 0.366 | 0.715 | 0.853 |
| Pfirrmann | LDD | 2.450 | (-9.229, 14.128) | 3.643 | 0.672 | 0.501 | 0.853 |
| Pfirrmann | MC | 1.220 | (-12.039, 14.478) | 4.136 | 0.295 | 0.768 | 0.853 |
| Bulge | Herniation | -4.932 | (-9.201, -0.662) | 2.178 | -2.264 | 0.024 | 0.118 |
| LDD | Herniation | -6.175 | (-10.376, -1.974) | 2.144 | -2.881 | 0.004 | **0.040** |
| MC | Herniation | -4.945 | (-11.242, 1.353) | 3.213 | -1.539 | 0.124 | 0.413 |
| Pfirrmann | Herniation | -3.725 | (-10.058, 2.608) | 3.231 | -1.153 | 0.249 | 0.622 |

Bivariate model post-hoc comparisons showing algorithms used to classify herniation had higher performance metrics than those classifying LDD. P-value adjustments were calculated using false discovery rate.

Confidence interval (CI), lumbar disc degeneration (LDD), Modic change (MC).
