# Supplementary material for: Are current machine learning applications comparable to radiologist classification of degenerate and herniated discs and Modic change? A systematic review and meta-analysis
Source: Eur Spine J. 2023 May 8;32(11):3764–87. doi: 10.1007/s00586-023-07718-0 (PMC10164619; doi:10.1007/s00586-023-07718-0)
Supplement: Supplementary file 11 — Supplementary file11 (DOCX 19 KB) [file 586_2023_7718_MOESM11_ESM.docx]

Supplementary Table 9 Risk of bias summary

| **Author – date** | **Standard participant details reported** | **All MRI specifications reported** | **Ethics approval stated** | **ROB introduced by participant selection** | **ROB introduced ground truth quality** | **Data augmentation?** | **Predictor assessments made blinded?** | **ROB introduced by the outcome or its determination** | **Participants excluded?** | **Model overfitting accounted for?** | **Risk of bias introduced by the analysis** | **Overall ROB judgement** |
| --- | --- | --- | --- | --- | --- | --- | --- | --- | --- | --- | --- | --- |
| Athertya, 2019 | No | Yes | No | Unclear | Unclear | Yes | PY | Low | Yes | PN | Low | Unclear |
| Athertya, 2021 | No | Yes | No | High | High | Yes | Yes | Low | No | PN | Unclear | High |
| Beulah, 2018 | No | Yes | No | Unclear | Low | No | Yes | Low | No | Yes | Low | Unclear |
| Beulah, 2021 | No | Yes | No | Unclear | Low | No | Yes | Unclear | Unclear | NI | Unclear | Unclear |
| Castro-Mateos, 2016 | Yes | Yes | Yes | Unclear | Low | No | Yes | Low | No | Yes | Low | Unclear |
| Ebrahimzadeh, 2018 | Yes | Yes | No | Unclear | Unclear | No | PN | High | No | NI | Low | High |
| Gao, 2021 | Yes | Yes | Yes | Low | Low | No | Yes | Low | Yes | Yes | Low | Low |
| Ghosh, 2011 | No | Yes | No | Low | Low | No | Yes | Low | No | Yes | Low | Low |
| Gong, 2021 | Yes | Yes | No | Low | Unclear | No | Yes | Unclear | Unclear | Unclear | Low | Unclear |
| Grob, 2022 | Yes | Yes | Yes | Low | Low | NA | Yes | Low | Yes | NA | Low | Low |
| Han, 2018 | Yes | Yes | No | Unclear | Unclear | No | PY | Unclear | Unclear | Yes | Unclear | Unclear |
| Hashia, 2020 | No | No | No | High | High | No | NI | Unclear | Unclear | NI | High | High |
| He, 2017 | Yes | Yes | No | Unclear | Unclear | No | PY | Low | Unclear | Yes | Low | Unclear |
| Jamaludin, 2016 | No | Yes | No | Low | Unclear | Yes | PY | Low | Yes | Yes | Low | Unclear |
| Jamaludin, 2017 | No | Yes | No | Low | Unclear | No | PY | Unclear | Yes | PY | Low | Unclear |
| Koh, 2012 | No | Yes | No | Unclear | Unclear | No | Yes | Unclear | Unclear | NI | Unclear | Unclear |
| **Author – date** | **Standard participant details reported** | **All MRI specifications reported** | **Ethics approval stated** | **ROB introduced by participant selection** | **ROB introduced ground truth quality** | **Data augmentation?** | **Predictor assessments made blinded?** | **ROB introduced by the outcome or its determination** | **Participants excluded?** | **Model overfitting accounted for?** | **Risk of bias introduced by the analysis** | **Overall ROB judgement** |
| Lehnen, 2021 | Yes | No | Yes | Low | Low | NA | Yes | Low | Yes | NA | Low | Low |
| Lewandrowski, 2020 | Yes | No | No | Low | Unclear | No | Yes | Unclear | No | Yes | Low | Unclear |
| Mc Sweeney, 2022 | No | Yes | Yes | Unclear | Low | NA | Yes | Low | Yes | NA | Low | Low |
| Niemeyer, 2021 | No | Yes | Yes | Low | Low | Yes | Yes | Low | Yes | Yes | Low | Low |
| Nikravan, 2016 | No | Yes | No | High | Unclear | No | No | Medium | Unspecified | NI | Unclear | High |
| Oktay, 2014 | No | Yes | No | Low | Unclear | No | Yes | Medium | No | PN | Unclear | Unclear |
| Pan, 2021 | No | Yes | No | Unclear | Low | No | Yes | Medium | No | No | Unclear | Unclear |
| Su,2022 | Yes | Yes | Yes | Low | Low | Yes | Yes | Low | Yes | PY | Low | Low |
| Sundarsingh, 2020 | No | Yes | No | Unclear | Unclear | No | No | Unclear | Unclear | PN | Unclear | Unclear |
| Tsai, 2021 | No | Yes | Yes | Unclear | Low | Yes | NI | Unclear | Unclear | Yes | Unclear | Unclear |
| Zheng, 2022 | No | Yes | Yes | Unclear | Low | No | Yes | Unclear | Yes | PY | Unclear | Unclear |
