# Supplementary material for: Are current machine learning applications comparable to radiologist classification of degenerate and herniated discs and Modic change? A systematic review and meta-analysis
Source: Eur Spine J. 2023 May 8;32(11):3764–87. doi: 10.1007/s00586-023-07718-0 (PMC10164619; doi:10.1007/s00586-023-07718-0)
Supplement: Supplementary file 12 — Supplementary file12 (DOCX 12 KB) [file 586_2023_7718_MOESM12_ESM.docx]

Supplementary 10. Github link to code

https://github.com/rogercompte/Systematic-review-ML-of-MRI-and-LDD
